# Intraovarian injection of autologous human mesenchymal stem cells increases estrogen production and reduces menopausal symptoms in women with premature ovarian failure: two case reports and a review of the literature

**DOI:** 10.1186/s13256-020-02426-5

**Published:** 2020-07-18

**Authors:** Prosper Igboeli, Abdeljabar El Andaloussi, Ujalla Sheikh, Hajra Takala, Amro ElSharoud, Ashley McHugh, Larisa Gavrilova-Jordan, Steven Levy, Ayman Al-Hendy

**Affiliations:** 1 Department of Obstetrics and Gynecology, University of Illinois at Chicago, 820 South Wood Street, Chicago, IL 60612 USA; 2 Department of Pathology, University of Illinois at Chicago, 820 South Wood Street, Chicago, IL 60612 USA; 3 Department of Obstetrics and Gynecology, Augusta University, Augusta, GA 30912 USA; 4 MD Stem Cells, Sylvan Road South, Westport, CT 06880 USA

**Keywords:** Premature ovarian failure, Infertility, Cell therapy, MSCs, BMSCs, Bone marrow–derived stem cells, Mesenchymal stem cells, Case report

## Abstract

**Background:**

Premature ovarian failure is a relatively common condition that affects 1–3% of adult women. Premature ovarian failure occurs when there is loss of ovarian function in women younger than 40 years of age. The causes are mostly iatrogenic or idiopathic. Amenorrhea and infertility are the most important clinical manifestations. So far, no therapeutic intervention has been proved effective in restoring fertility in patients with premature ovarian failure. Attempts to stimulate ovarian function through hormone manipulation typically prove unsuccessful, and patients usually resort to egg donation to achieve pregnancy. In our preclinical work, intraovarian administration of human bone marrow–derived mesenchymal stem cells was able to restore ovarian hormone production, reactivate folliculogenesis, and reverse infertility in a chemotherapy-induced ovarian failure mouse model.

**Case presentation:**

We present two cases of Caucasian women with premature ovarian failure who resumed ovarian estrogen production and menses 7 months following autologous bone marrow–derived mesenchymal stem cell injections into the ovary. This pilot clinical study is registered with ClinicalTrials.gov (identifier NCT02696889). In this report, we present data from our first two cases that have completed study procedures so far. The bone marrow–derived mesenchymal stem cells were harvested from the bone marrow of the iliac crest of the patients with premature ovarian failure and nucleated cells concentrated and enriched in bone marrow–derived mesenchymal stem cells intraoperatively, and then injected into the patient’s right ovary via laparoscopy. Autologous bone marrow stem cell engraftment into the ovary resulted in several improvements in the treated patients with premature ovarian failure. In measurements by transvaginal ultrasound, there were increases of approximately 50% in volume of the treated ovaries in comparison with the contralateral control ovaries that persisted to the end of the study (1 year). Serum levels of estrogen increased by approximately 150% compared with the preoperative levels. Each of the two patients had an episode of menses, and also both of them reported marked improvement of their menopausal symptoms that also persisted to the end of the study (1 year). The bone marrow–derived mesenchymal stem cell implantation procedure was very well tolerated with no reported adverse events.

**Conclusions:**

Our study reveals promising improvement of premature ovarian failure–related clinical manifestations in two patients after intraovarian autologous bone marrow–derived mesenchymal stem cells engraftment. These early observations call for additional assessment and further development of intraovarian bone marrow–derived mesenchymal stem cell injection for possible treatment of patients with premature ovarian failure.

## Background

Premature ovarian failure (POF), also called *premature ovarian insufficiency*, is a challenging reproductive issue causing loss of ovarian function in women younger than 40 years of age [1–5]. Despite intense research, the causes of POF remain a mystery in most cases [3, 6]. POF is characterized by hypoestrogenism; increased serum gonadotropin levels; and, most important, amenorrhea [1, 3, 5, 6]. POF affects about 1–3% of women in the general population [7], but its prevalence is up to 10–28% in females with primary amenorrhea and about 4–18% in women with secondary amenorrhea [7]. POF is a devastating diagnosis for women of reproductive age [2]. Ovaries of patients with POF are characterized by loss of secondary follicles and arrested folliculogenesis, which leads to a decrease or elimination of estrogen production and infertility. The mechanism of the ovarian follicles’ depletion in patients with POF is most likely an accelerated follicular atresia, but the detailed pathogenesis is not fully understood yet [8]. In addition, estrogens are essential for normal folliculogenesis beyond the antral stage [9] and are known to be decreased in patients with POF. Also, POF is associated with serious health consequences, including psychological distress, osteoporosis, autoimmune disorders, ischemic heart disease, Alzheimer disease, metabolic syndrome, diabetes, and increased risk of mortality [2].

For patients with POF who desire pregnancy, the only current option is to receive donor eggs. Recently, several lines of preclinical evidence suggest that cell therapy (CT) using bone marrow–derived mesenchymal stem cells (BMSCs) [10] can restore the structure and function of injured ovarian tissues [11–14]. CT is based on groundbreaking scientific discoveries and technological advancements. Most cell-based therapies are currently experimental, with a few exceptions [10]. CT is defined as the transfer or engraftment of living cells into damaged tissue [15]. CTs can be classified mainly by the origin of cells (autologous or allogeneic transplant) or by their treatment indications (for example, hematological, reproductive, or neurological pathologies). The most common type of CT is the replacement of mature cells through blood and platelet transfusions. Other potential therapeutic applications of CT include treating urinary problems or infectious diseases; rebuilding damaged cartilage in joints; and helping patients with neurological disorders, brain or spinal cord injuries, heart disease, diabetes [16], and, more recently, cancer [17].

Human stem cells play a key role and have important potential application in cell-based therapies that represent a possible alternative for a variety of diseases, including reproductive dysfunction. Our preclinical study of POF animal models treated with human BMSCs showed that BMSC-treated animals recovered ovarian function, resulting in folliculogenesis and estrogen production [13, 18]. In another study, using cisplatin to induce granulosa cell apoptosis, the authors demonstrated that the apoptosis was reduced by BMSCs *in vitro* [19]. In addition, the MSC treatment group showed increased antral follicle count and estradiol (E2) after 1 month compared with the untreated POF group [20]. According to these results, BMSCs may potentially revive prematurely failed ovaries in both follicular and hormonal aspects. The role of stem cells in treating diseases is related to their unique regenerative abilities, giving rise to different cells and tissues [21].

Stem cell therapies can be applied directly, as in the context of bone marrow transplantation, or by the use of more or less mature cells produced *in vitro* from stem cells. Today, donated cells, tissues, and organs from healthy donors are often used to replace diseased or destroyed tissue [22], but in certain cases, such as in POF, some patients are unwilling to choose this option. Thus, there is a critical need to develop novel effective approaches for POF treatment. In this study, we report early observations of the biological effect of BMSCs as a possible therapeutic tool on the phenotype of POF.

## Cases

Patients were recruited into the study according to the following inclusion criteria: age over 18, primary or secondary amenorrhea at least for 6 months, at least two menopausal follicle-stimulating hormone (FSH) levels (> 40 IU/L), normal karyotype 46,XX, and presence of at least one ovary. The study was approved by the Augusta University Institutional Review Board (no. 723327-2), and study procedures were initiated only after patients signed informed consent. The inclusion and exclusion criteria are summarized in Table 1. In addition, the hormone levels prior to and 12 months after mesenchymal stem cell injection into the right ovary are summurized in Table 2.

**Table 1:** Summary of inclusion and exclusion criteria

Inclusion	Exclusion
1. Over the age of 18 2. Diagnosis of POF (two menopausal FSH levels > 40 IU/L or primary or secondary amenorrhea at least for 3–6 months) 3. Normal karyotype 46,XX 4. At least one ovary 5. At least unilateral tubal patency 6. Acceptable uterine anatomy 7. No male infertility 8. Normal thyroid function 9. Planning and/or willing to attempt to become pregnant as part of the experiment 10. No other causes of female infertility	1. Currently pregnant or breastfeeding 2. History or evidence of current gynecologic malignancy within the past 3 years 3. Presence of adnexal masses 4. Major mental health disorder 5. Active substance abuse or dependence 6. Unfit/unwilling to undergo laparoscopy 7. Medical conditions that are contraindicated in pregnancy

*FSH* Follicle-stimulating hormone, *POF* Premature ovarian failure

**Table 2:** Hormone levels prior to and 12 months after mesenchymal stem cell injection into the right ovary

	Reference range	Preoperative		1 week		1 month		3 months		6 months		9 months		12 months	
		P1	P2	P1	P2	P1	P2	P1	P2	P1	P2	P1	P2	P1	P2
AMH	(1.1–53.5 ng/ml)	< 0.0140	< 0.015	0.0140	< 0.015	< 0.0140	< 0.015	< 0.014	< 0.015	< 0.1	*	< 0.0140	< 0.015	< 0.1	*
FSH	(3.0–8.0 IU/L)	89.4	150.8	132	161.4	91.2	149.9	78.1	147.1	68.5	*	89.2	150.1	80.3	*
LH	(2.0–12.0 IU/L)	45.8	67.0	48.3	69.5	36.3	75.2	37.3	67.8	41.4	*	45.0	71.0	41.3	*
CA 125	< 36 kU/L	11	10.2	13	20.6	13	12.0	11	10.6	2.9	*		12.3		*
Estradiol	(27–123 pg/ml)	< 10.896	*	19.88	< 5.0	14.165	< 5.0	< 10.9	< 5.0	26.15	*	21.79	10.9	19.3	*
Progesterone	(0.1–0.7 ng/ml)	0.1887	0.5		0.2	0.2201	< 0.1	< 0.16	< 0.1	< 0.157	*	0.2201	< 0.1	*	*

*Abbreviations: AMH* Anti-Müllerian hormone, *CA 125* Cancer antigen 125, *FSH* Follicle-stimulating hormone, *LH* Luteinizing hormone, *P1* Patient #1, *P2* Patient #2

*Data not available

### First case

A 36-year-old Caucasian woman presented with secondary amenorrhea of 4 years’ duration. She is a pediatric dentist from the Toronto area in Canada. She was married in 2009 and immediately started using combined oral contraceptive pills for 3 years to delay childbearing. After she stopped taking oral contraceptive pills in January 2012 to attempt pregnancy, her periods never came back, and she had amenorrhea. Her Reproductive Endocrinology and Infertility Physician (REI) diagnosed her with POF FSH (110 IU/L). Since then, she had been treated with hormone replacement therapy (HRT) on and off to control menopausal symptoms. She stopped any external hormones in March 2016 and was only using prenatal vitamins as supplements. She is gravida 0, para 0 (G0 P0). She had menarche at the age of 11. Her menses were regular every 28 days. She denies a history of sexually transmitted disease. She has no other chronic medical conditions, and her past surgical history includes only wisdom teeth removal. She has no known allergies, and she denies use of tobacco, alcohol, or other drugs. Her family history includes only a father with diabetes. Her physical examination showed Tanner stage V with normal anatomy. The complete results of her physical examination are listed in Table 3. The diagnosis of POF was confirmed in May 2012 according to laboratory tests that revealed persistently high serum FSH (110 IU/L) and luteinizing hormone (LH) (53.89 IU/L), as well as low E2 (< 10 pg/ml). She has a normal karyotype (46,XX) and a negative genetic test result for fragile X syndrome (*FMR1*). She was placed on dehydroepiandrosterone for approximately 6 months, then a follow-up ultrasound examination in July 2013 revealed no antral follicles. She started experiencing menopausal symptoms (hot flushes and vaginal dryness) and received on-and-off HRT for relief from her primary care physician in August 2013. Very rarely, they have found one or two follicles during the cycles, but she did not ovulate. She desired treatment for infertility but refused egg donation for religious reasons. At admission, all her routine preoperative laboratory test results were normal, as listed in Table 3.

**Table 3:** Summary of physical examination and laboratory test results at admission

	First case	Second case
Physical examination	General: Alert and oriented, no acute distress Respiratory: Lungs are clear to auscultation, respirations are nonlabored, breath sounds are equal, symmetrical chest wall expansion Cardiovascular: Normal rate, regular rhythm, no murmur, no gallop, good pulses equal in all extremities Breast: No mass, no tenderness, no discharge Gastrointestinal: Soft, nontender, nondistended, normal bowel sounds Genitourinary: Bimanual exam, speculum exam, and external genitalia all WNL Musculoskeletal Normal range of motion Normal strength No tenderness No swelling Integumentary: Warm, dry, pink Neurologic: Alert, oriented, no focal deficits Psychiatric: Cooperative, appropriate mood and affect, normal judgment	General examination: No acute distress Eye: Extraocular movements are intact, normal conjunctiva HENT: Normocephalic, normal hearing Respiratory: Lungs are clear to auscultation, respirations are nonlabored Cardiovascular: Normal rate, normal rhythm Gastrointestinal: Soft, nondistended Musculoskeletal: Normal range of motion, normal strength Integumentary: Warm, dry, no rash Neurologic: Alert, oriented Psychiatric: Cooperative, appropriate mood and affect Abdominal exam: large midline incision, several small laparoscopic incisions Pelvic exam: External genitalia WNL Speculum exam: WNL, small pyknotic external os Bimanual exam: WNL
Hematology	WBC 7.0 × 10^3^/mm^3^	WBC 5.0 × 10^3^/mm^3^
	RBC 4.73 × 10^6^/mm^3^	RBC 4.37 × 10^6^/mm^3^
	Hgb 14.1 g/dl	Hgb 12.5 g/dl
	Hct 42.3%	Hct 37.4%
	MCV 89.4 fl	MCV 85.7 fl
	MCH 29.7 pg	MCH 28.5 pg
	MCHC 33.3 g/dl	MCHC 33.3 g/dl
	RDW 12.7%	RDW 13.8%
	Platelets 224 × 10^3^/mm^3^	Platelets 166 × 10^3^/mm^3^
	MPV 9.4 fl	MPV 10.0 fl
	Prothrombin time 10.9 seconds	Prothrombin time 12.0 seconds
	INR 0.9	INR 1.0
	APTT 35.2 seconds	APTT 29.8 seconds
	Urine pregnancy test negative	Urine pregnancy test negative

*Abbreviations: APTT* Activated partial thromboplastin time, *Hct* Hematocrit, *Hgb* Hemoglobin, *INR* International normalized ratio, *MCH* Mean corpuscular hemoglobin, *MCHC* Mean corpuscular hemoglobin concentration, *MCV* Mean corpuscular volume, *MPV* Mean platelet volume, *RBC* Red blood cells, *RDW* Red blood cell distribution width, *WBC* White blood cells, *WNL* Within Normal Limits, *HENT* Head, Ears, Nose, Throat

### Second case

A 42-year-old Caucasian woman, G0 P0, presented with secondary amenorrhea. She is a scientist with a doctoral degree at Temple University in Philadelphia with a focus on chronic obstructive pulmonary disease. She presented with high FSH (155 IU/L) and a normal karyotype (46,XX). She had had normal regular menses until 2014. Then, she was diagnosed with a large left ovarian cyst and needed surgery. She underwent robotic laparoscopy, but this had to be changed to midline open laparotomy, and she underwent left oophorectomy and also a myomectomy. After surgery, she had secondary amenorrhea, and she was then diagnosed with POF. She has experienced some menopausal symptoms but denied using any medication for these. She and her husband have been actively trying to achieve a pregnancy through regular intercourse but have not attempted any additional fertility treatments. She had normal hysterosalpingogram and a normal right ovary by ultrasound. Her past medical history only included occasional low blood pressure and remote tonsillectomy. She has no known allergies, and she denies use of tobacco, alcohol, or other drugs. She had a normal physical examination and laboratory workup, the results of which are listed in Table 3. The patient sought infertility treatment but refused egg donation for cultural reasons. She and the patient in case 1 both fit the criteria for inclusion in the present study according to the screening of their medical, surgical, and social histories and physical examinations.

## Methods

### Preparation of autologous bone marrow MSC-derived stem cells

With the patients under general anesthesia, the procedure involved the collection of bone marrow tissue from the iliac bone (posterior iliac crest) using a bone marrow aspiration kit soaked in heparin (BD Biosciences, Franklin Lakes, NJ, United States of America (USA)). About 150 ml of marrow tissue was harvested and subjected to concentration. We used a U.S. Food and Drug Administration–cleared class 2 medical Angel device (Arthrex Inc., Naples, FL, USA) that allowed us to concentrate 160 ml of bone marrow aspirate in about 20 minutes. The collected cell pellet was resuspended in 4 ml of bone marrow nucleated cell concentrate (~ 5 × 10^8^ total nucleated cells and an average of 5–15 million MSCs).

### Laparoscopy and direct injection of right ovary

**Fig. 1:**
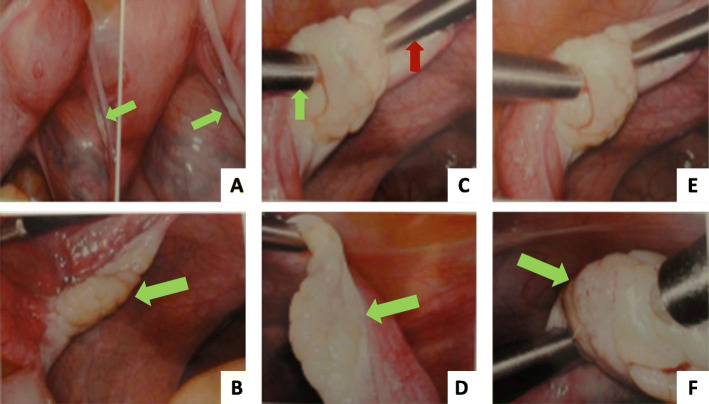
Ovarian morphologic changes after mesenchymal stem cell injection. **a** Atrophic ovary before injection (*green arrows*) (low magnification). **b** Atrophic ovary before injection (*green arrow*) (zoom-in). **c** Laparoscopic grasper (*green arrow*) to stabilize the ovary during injection process via stem cell injector (*red arrow*). **d** Atrophic ovary (*green arrow*) stabilized in place just before injection process. **e** Injection of the stem cells. **f** Ovary (*green arrow*) near end of injection process (Notice the swelling and apparent increase in ovarian size.)

We used laparoscopy for direct injection of the harvested BMSCs (4 ml) into the right ovary. A similar amount (4 ml) of normal saline was injected into the left ovary to serve as a control. In case 2, this was not done, because the patient had undergone prior left oophorectomy. Injection was conducted using a 5-mm injector (22-gauge needle). An assistant firmly stabilized the ovary using an atraumatic grasper (Fig. 1). The surgeon inserted the needle via one entry through the ovarian capsule toward the center of the ovary and slowly injected the cell concentrate over a 5-minute time period to allow gradual expansion of the ovary. After all solution was injected, the needle was left *in situ* for another 5 minutes, acting as a stopper to avoid backflow of injected cells out of the ovary.

### Transvaginal ultrasound imaging

After MSC engraftment, a standard transvaginal ultrasound technique was used for the measurement of endometrial thickness, ovarian volume, and antral follicle count (AFC) at 1 week, 1 month, 3 months, 6 months, 9 months, and 12 months.

### Biochemical evaluation of serum hormone levels and symptom evaluation

Serum hormone profiles were checked for 1 year postengraftment at 1 week and 1, 3, 6, 9, and 12 months. The examined hormones included FSH, LH, anti-Müllerian hormone (AMH), E2, and progesterone. The serum hormone quantification was done by radioimmunoassay using standard laboratory techniques [23]. Symptoms were self-reported by both patients to the study coordinators.

## Results

**Fig. 2:**
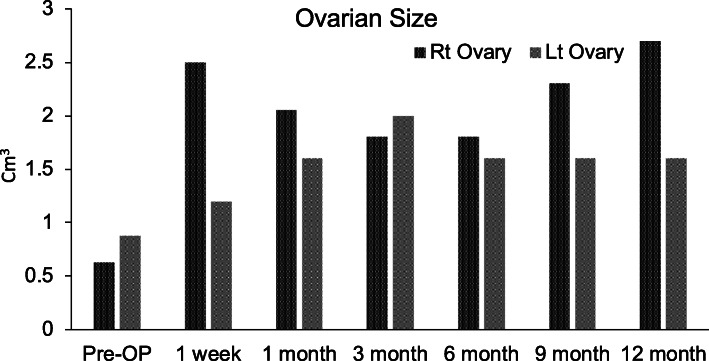
Ovarian volume measured in cubic centimeters after mesenchymal stem cell implantation into ovary of patient 1

**Fig. 3:**
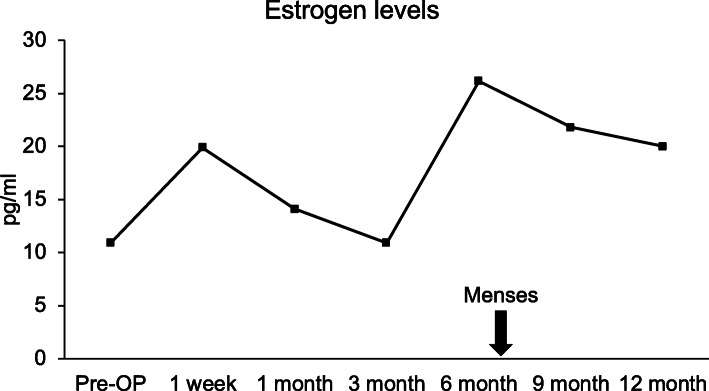
Serum estrogen level quantified at pre– and post–mesenchymal stem cell ovarian implantation. The *arrow* indicates the menses that resume within 7 months following mesenchymal stem cell engraftment

**Fig. 4:**
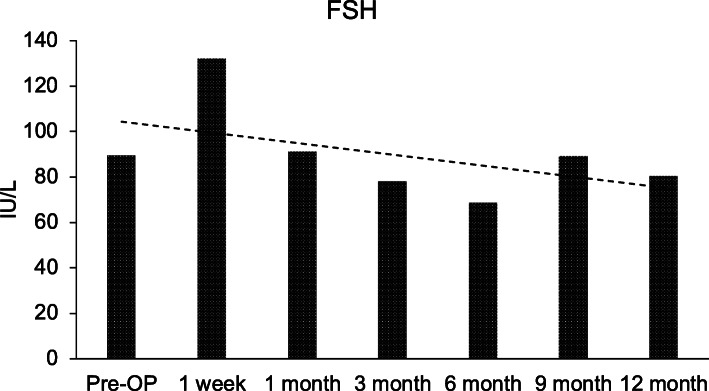
Serum follicle-stimulating hormone (FSH) level reported pre– and post–mesenchymal stem cell ovarian implantation

**Fig. 5:**
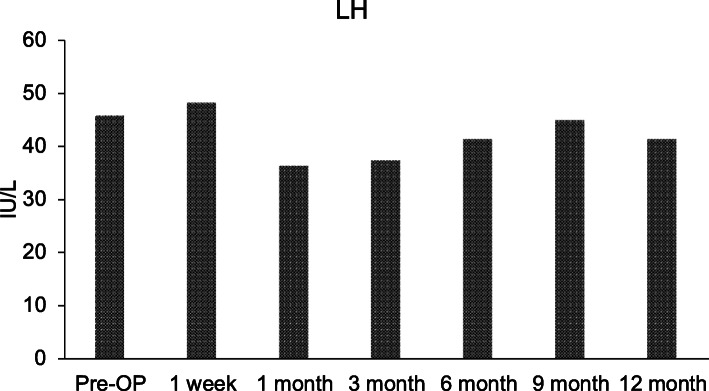
Serum luteinizing hormone (LH) level examined pre– and post–mesenchymal stem cell ovarian implantation

Autologous BMSCs were injected laparoscopically into the right ovary. The left ovary (in the first case) was subjected to sham surgery for control (Fig. 1). After injection, the volume of the right ovary exhibited a significant increase in size, confirming proper injection and cell delivery inside the ovary. Ovarian volume and hormone levels were monitored at various time points per study protocol; however, several data points were missing in the second case because of noncompliance. Figures 2, 3, 4 and 5 are from the first case.

### First case

Ovarian volume of the test ovary increased from 0.63 cm^3^ before injection to 2.5 cm^3^ at week 1 of follow-up, then 2.05 cm^3^ 3 weeks later. Follow-up at months 3 and 6 displayed stability in ovarian volume, measuring at 2.3 cm^3^ and 2.7 cm^3^, respectively, with an increase in size after regaining menses at 7 months (Fig. 2). These data suggest that the right ovary responded specifically to the BMSC injection *in situ*. Furthermore, we noticed an overall increase in serum estrogen level after BMSC engraftment (Fig. 3). The estrogen level was < 10.9 pg/ml prior to MSC implantation and 1 week after MSC implantation, and it increased by three times to 20 pg/ml (Fig. 3). At 6 months after MSC injection, estrogen level reached of 26.1 pg/ml. There were also changes in serum levels of FSH and LH, as shown in Figs. 4 and 5, respectively, but no changes were observed in serum progesterone levels, anti-Müllerian hormone levels, or antral follicle counts after BMSC implantation compared with preprocedural levels.

### Second case

We observed an increase in estrogen levels at 9-month follow-up. There were no changes in the FSH, LH, or progesterone levels. Unfortunately, because of this patient’s noncompliance, we are missing several data on serum hormone panels. A decrease of menopausal symptoms, such as hot flushes, night sweats, and painful intercourse, was reported by both patients. Both patients reported one incident of menses (3 days of vaginal bleeding, mild to moderate in amount) at 7 months after BMSC implantation.

## Discussion

POF is a devastating medical condition with currently very limited treatment options, which usually fail to address the patients’ issues and needs. POF interferes with fertility and causes menopausal symptoms as well as serious psychological sequelae [24]. All together, these challenges require a multidisciplinary approach in diagnosis and treatment as well as special attention. The current established modalities for treating POF mainly target the hypoestrogenism-related symptoms by using external HRT [25]. There are no controlled studies regarding the ideal hormone replacement strategy for young women with spontaneous POF [26]. The absence of evidence-based guidelines regarding the ideal hormone replacement strategy for young women with POF poses a clear challenge.

Spontaneous resumption of ovarian activity is possible in women with POF, indicating that this condition is not irreversible and is likely due to the presence of residual oocytes in these so-called failed ovaries. Women may conceive spontaneously or following different regimens of ovulation induction, thus indicating that ovarian failure is not always permanent [27]. Recently, *in vitro* maturation (IVM) using ovarian fragmentation combined with Akt stimulation treatment to disrupt ovarian Hippo signaling was developed. A viable birth was reported using IVM followed by *in vitro* fertilization (IVF)-embryo transfer [28]. In a clinical trial including 14 patients with POF, follicle development waves were detected in 43% of the examined patients. After IVF of retrieved oocytes, four early embryos were derived; one patient became pregnant and delivered a healthy baby boy after embryo transfer [29]. In another study, ovarian tissue cryopreservation using verification followed by IVM was done for infertility management in 37 patients with POF. The reported results of this study showed that 54% of women had residual follicles and 9 of 20 showed follicular growth with 24 oocytes in autografts retrieved from 6 patients. Following IVF and embryo transfer into four patients, three chemical pregnancies were detected, followed by one miscarriage and two successful deliveries [30]. This method has multiple limitations, including multiple surgical interventions: one for the collection of the ovarian tissue and another for implantation after IVM and the additional IVF protocols and procedures.

Due to the ability of BMSCs in restoring physiological function of many organs, there are 344 registered clinical trials trying to evaluate the potential of BMSC-based CT against a plethora of human diseases worldwide. BMSCs have been shown to be effective in the treatment of many diseases with the advancement of preclinical studies [31]. Furthermore, MSCs have been shown in almost all tissues. They can be easily isolated from the bone marrow, adipose tissue, umbilical cord, fetal liver, muscle, and lung and can be successfully expanded *in vitro* [32]. It is better to use freshly isolated MSCs in stem cell therapy, because it has been shown that major histocompatibility complex II molecules could be increased during *in vitro* expansion of the stem cells, which may increase their allogenicity [33]. Recent studies have suggested that the allogenicity of MSCs has no significant adverse impact on their engraftment in wound healing, though [34]. Multiple studies have also demonstrated the ability of stem cell bioactive factors (the secretome) to restore the ovarian function and regeneration [35–37]. Earlier research primarily attributed the effects of the MSC potential therapy to these cells’ capacity for local differentiation into various tissue types. However, recently, research studies have indicated that implanted MSCs have short lifespans, and their therapeutic benefits could be due to their secretome [38]. The secretome is formed of molecules secreted into the extracellular space and consists of soluble proteins, free nucleic acids, lipids, and extracellular vesicles. These vesicles include apoptotic bodies, microparticles, and exosomes. Gu *et al.* studied the reparative effects of MSCs on vascular tissues. They found that two mechanisms are involved in vascular regeneration: the MSCs’ multipotent differentiation ability to produce endothelial cells, vascular smooth muscle cells, and other cell types, as well as their capacity to secrete various trophic factors. These factors are potent in promoting angiogenesis, inhibiting apoptosis, and modulating immunoreactions [39].

The immunomodulatory effect of MSCs works through immune cells such as natural killer (NK) cells, B and T cells, and dendritic cell (DC) differentiation and migration [40]. Additionally, coculture of the MSC secretome promotes the anti-inflammatory phenotype of DCs, T cells, macrophages, and NK cells [41, 42]. In addition, the MSC secretome includes a number of molecules, such as prostaglandin E_2_, tumor necrosis factor-α-stimulated gene-6, transforming growth factor-β, hepatocyte growth factor (HGF), and interleukin-10, which may mediate the immunomodulatory function of MSCs [43, 44]. In the context of POF, the regenerative mechanism of BMSCs in the ovary could be mainly via the promotion of the angiogenesis. Like in other reported models, MSCs produce a large variety of humoral factors that may play a role in tubular formation by recruiting ovarian endothelial microvessel cells [45, 46]. The data from our experiments in our lab suggest that the MSC secretome can produce similar effects on human granulosa cells *in vitro* [47]. In a recent publication by our group, Park *et al.* reported that the expression of proliferation marker Ki67 was significantly increased by treatment with the MSC secretome in human ovarian endothelial cells [48]. MSC secretome treatment also induced significantly higher expression of several angiogenic markers, such as vascular endothelial growth factor (VEGF) receptor 2, Tie2/Tek, VE-cadherin, endoglin, and VEGF, than of matched controls. It suggests that the MSC secretome likely contains bioactive factors that can enhance ovarian angiogenesis. Other studies have indicated that BMSCs are able to differentiate into endothelial cells, pericytes, or even vessel walls to support the formation of blood vessels [49]. Furthermore, it was suggested that MSCs are also capable of protecting endothelial cells from apoptosis, including oxidative stress–related apoptosis in the initial phase of angiogenesis [50]. The role of MSCs in promoting angiogenesis was reported in various studies demonstrating that MSCs support the late phases of angiogenesis, including blood vessel maturation [51, 52]. This is also consistent with our preclinical work where estrogen-responsive organs demonstrated remarkable increases in the mean weight, such as for the ovaries, uterus, kidneys, and liver [13]. Also, multiple studies demonstrate that transplanted MSCs may play an important role in the ovarian microenvironment paracrine regulation, producing a wide array of cytokines, such as VEGF, insulin-like growth factor-1, and HGF, that inhibit apoptosis [53].

The results of the first case show that the use of autologous BMSCs injected into the ovaries of patients with POF has a measurable/quantifiable effect with promising results that may make this method a potential treatment option for these patients in the future. In our study, the patients underwent BMSC collection and injection in the same setting while under general anesthesia, which is widely acceptable to patients interested in this trial. In a similar study using MSCs to treat POF, researchers claimed that the use of a similar protocol in patients with POF resulted in spontaneous pregnancy in one patient, but the MSC preparation details, post-procedural serum hormone levels, and imaging data were lacking from that brief report [54].

Our data show an immediate impact on ovarian volume that persisted throughout the following assessments. There was a consistent and sustained increase in serum estrogen levels through follow-up at 1 week and 1, 3, 6, and 9 months. Such partial correction of a hypoestrogenic state may explain the patient-reported reversal of several menopausal symptoms particularly noticed about 3 months after MSC implantation and onward. Interestingly, there was no increase in the volume of the left control ovary. Both patients reported one incident of spontaneous menses about 7 months after BMSC implantation after several years of absolute amenorrhea, potentially due to the partial correction of E2 hormone levels. Because there was no increase in serum progesterone levels and transvaginal ultrasound (TVS) revealed no detectable antral follicles, one assumes that this anovulatory menstrual bleeding is likely due to the effect of increasing serum estrogen levels on the endometrium after BMSC implantation. These results can be due to the effect of MSCs on the regeneration of the granulosa cells and initiation of folliculogenesis likely up to the secondary follicle stage, which may explain the increase in serum E2 levels with no change in AFC. The increase in size of the right (treated) ovary versus pretreatment size and also compared with the left (control) ovary suggests resumption of folliculogenesis as well as likely stimulated angiogenesis in these injected ovaries. Neither patient seemed to achieve ovulation or reported pregnancies during the 1-year study period. This can be due to a number of factors; for example, the 12-month duration of this trial may not be sufficient to restart the long-stagnant folliculogenesis. Another reason may be the use of autologous MSCs, because several reports have suggested that autologous MSCs from patients with systemic diseases may be suboptimal and possess inferior regenerative abilities compared with such cells from healthy donors [55–57]. Although these early observations are encouraging, we recognize the limitation of our report due to a small number of cases and the lack of several data points from the second case. Our study is active and registered with ClinicalTrials.gov (www.clinicaltrials.gov/ct2/show/NCT02696889) with additional enrollment. Additional research in this area is urgently needed to fully explore the potential of MSCs as a promising treatment for this devastating disease.

## Conclusion

Our study reveals encouraging preliminary data observed after autologous BMSC engraftment into the ovaries of patients with POF. The BMSC delivery into the ovary diminished menopausal symptoms observed at preoperative assessments. The treated patients were able to resume menses within 7 months following MSC engraftment. In addition, no complications or safety concerns were reported in our study. More studies are needed to evaluate this approach, and we will also continue reporting our ongoing clinical trial.

## Data Availability

Our study is active and is registered with ClinicalTrials.gov (https://www.clinicaltrials.gov/ct2/show/NCT02696889).

## Abbreviations

AFC: Antral follicle count
AMH: Anti-Müllerian hormone
APTT: Activated partial thromboplastin time
BMSC: Bone marrow–derived stem cell
CA 125: Cancer antigen 125
CT: Cell therapy
DC: Dendritic cell
E2: Estradiol
FSH: Follicle-stimulating hormone
Hct: Hematocrit
Hgb: Hemoglobin
HGF: Hepatocyte growth factor
HRT: Hormone replacement therapy
INR: International normalized ratio
IVF: *In vitro* fertilization
IVM: *In vitro* maturation
LH: Luteinizing hormone
MCH: Mean corpuscular hemoglobin
MCHC: Mean corpuscular hemoglobin concentration
MCV: Mean corpuscular volume
MPV: Mean platelet volume
NK: Natural killer
POF: Premature ovarian failure
RBC: Red blood cells
RDW: Red blood cell distribution width
VEGF: Vascular endothelial growth factor
WBC: White blood cells
